# Age dependent normal horizontal VOR gain of head impulse test as measured with video-oculography

**DOI:** 10.1186/s40463-015-0081-7

**Published:** 2015-07-04

**Authors:** Benjamin Mossman, Stuart Mossman, Gordon Purdie, Erich Schneider

**Affiliations:** Department of Neurology, Wellington Hospital, Riddiford Street, Private Bag 7902, Wellington South, Wellington New Zealand; Dean’s Department, University of Otago, Wellington, New Zealand; Institute of Medical Technology, Brandenburg University of Technology Cottbus, Senftenberg, Germany

**Keywords:** Head impulse test, Horizontal vestibulo-ocular reflex, Semicircular canal, Eye movements

## Abstract

**Background:**

The head impulse test (HIT) is a recognised clinical sign of the high frequency vestibulo-ocular reflex (VOR), which can be quantified with video-oculography. This measures the VOR gain as the ratio of angular eye velocity to angular head velocity. Although normative data is available for VOR gain with video-oculography, most normal studies in general include small numbers of subjects and do not include analysis of variation of VOR gain with age. The purpose of our study was to establish normative data across 60 control subjects aged 20 to 80 years to represent a population distribution.

**Methods:**

Sixty control subjects without any current or previous form of brain disorder or vertigo participated in this study and form the basis for future comparison to patients with vestibular lesions. The relationship between the horizontal vestibulo-ocular reflex (HVOR) velocity gain and age was analysed using a mixed regression model with a random effect for subjects. Differences in testing technique were assessed to ensure reliability in results.

**Results:**

The mean HVOR velocity gain of 60 normal subjects was 0.97 (SD = 0.09) at 80 ms and 0.94 (SD = 0.10) at 60 ms. The 2 SD lower limit of normal HVOR velocity gain was 0.79 at 80 ms and 0.75 at 60 ms. No HVOR velocity gain fell below 0.76 and 0.65 at 80 ms and 60 ms respectively. The HVOR velocity gain declined by 0.012 and 0.017 per decade as age increased at 80 ms and 60 ms respectively. A non-physiologically high horizontal HVOR velocity gain was found to occur in tests where passive HITs were predictable in direction and time and where target distance was below 0.70 m.

**Conclusions:**

Normative data with respect to HVOR velocity gain decreases slightly with age, but with careful attention to methodology the 2 SD lower limit of normal is relatively robust across a wide age range and into the eighth decade, without requirement for adjustment with age.

## Background

The horizontal head impulse test (HIT) is a well recognised clinical tool to test the horizontal vestibular ocular reflex (HVOR). The subject maintains fixation on an object straight ahead while sudden head impulses are applied in the horizontal angular plane and eye movements are observed for catch up saccades [1]. If a subject’s vestibular ocular reflex (VOR) is normal, the eyes should remain focused on the fixation target during head rotation. However, if there is a significant semicircular canal deficit on the side corresponding to rotation, the ipsilateral VOR response will be inadequate and a significant catch up saccade(s) may be seen.

Video-oculography (VOG) goggles allow quantitative recording of the eye and head movements during the HIT. Covert saccades (occurring during the head movement, usually undetectable clinically) and overt saccades (occurring after the head movement and detectable clinically) are both recorded by the camera. VOG also provides a quantitative measure of the VOR deficit, distinguishing abnormal from normal subjects. An advantage of the video HIT (vHIT) over search coils [2] is that it is less invasive, has simple setup and is readily available for clinicians. This relatively new technology uses a high speed compact camera (220 Hz sampling rate) that is attached to lightweight goggles (EyeSeeCam HIT) [3]. The system is used in conjunction with computer software to track pupil movement. Head movement is recorded through a motion sensor attached to the goggles [3–5]. A velocity gain is calculated by dividing instantaneous eye velocity by instantaneous head velocity. The objective was to obtain HVOR velocity gain data to represent a population distribution of normal subjects.

## Methods

The study used quantitative recordings with VOG to measure the HVOR velocity gain during the high frequency horizontal HIT. Sixty three normal subjects were tested (10 per decade, ranging from 20 to 80 years). Exclusion criteria, ascertained at the time of recruitment were no previous form of brain disorder, vertigo, or restricted neck movement.

Before testing, each subject was given verbal and written information regarding the test procedure and rationale. This outlined the risks and exclusion criteria, with signed consent required. As the study established normative data, approval was not required by our Central Regional Ethics Committee.

### Experimental procedure

Before formal testing, we ensured that the subject managed an adequate range of unrestricted, painless angular head rotation. The subject was seated 1.5 m directly in front of a fixation target at eye level. VOG goggles were fitted tightly to the subject’s head to reduce goggle slippage. The camera was focused on the eye while the subject fixated on the target. The subject was instructed to keep his/her eyes open widely so as not to obscure the pupil. If the palpebral fissure remained unduly narrowed, including from ptosis with redundant skin folds or long eye lashes, the eyelids were held open by the rims of the goggles. Even though this procedure can alter the vertical offset of the calibration parameters, it has no effect on the scaling of the calibration. The HVOR velocity gain therefore remains unaffected.

The system was calibrated with the subject altering fixation around five dots, 8.5° apart, projected onto the wall in front of them. The dots were emitted from a goggle-mounted laser and a diffraction grating [6]. The fixation sequence was arbitrary and the subject was instructed to spend no more than one second fixating on each dot. If errors occurred, the operator could repeat the calibration procedure.

The testing method outlined to the subjects included that they should:

- clench their teeth during the HIT to reduce jaw movement and facilitate a more direct force transfer to the head and reduce movement artefact
- maintain a relaxed neck musculature and not anticipate or aid in head movements
- not move the goggles once calibration was completed
- keep their eyes open wide and minimise blinking to allow the software to keep precise track of pupil movements
- maintain gaze on the fixation target throughout the testing procedure of angular head rotation

**Fig. 1:**
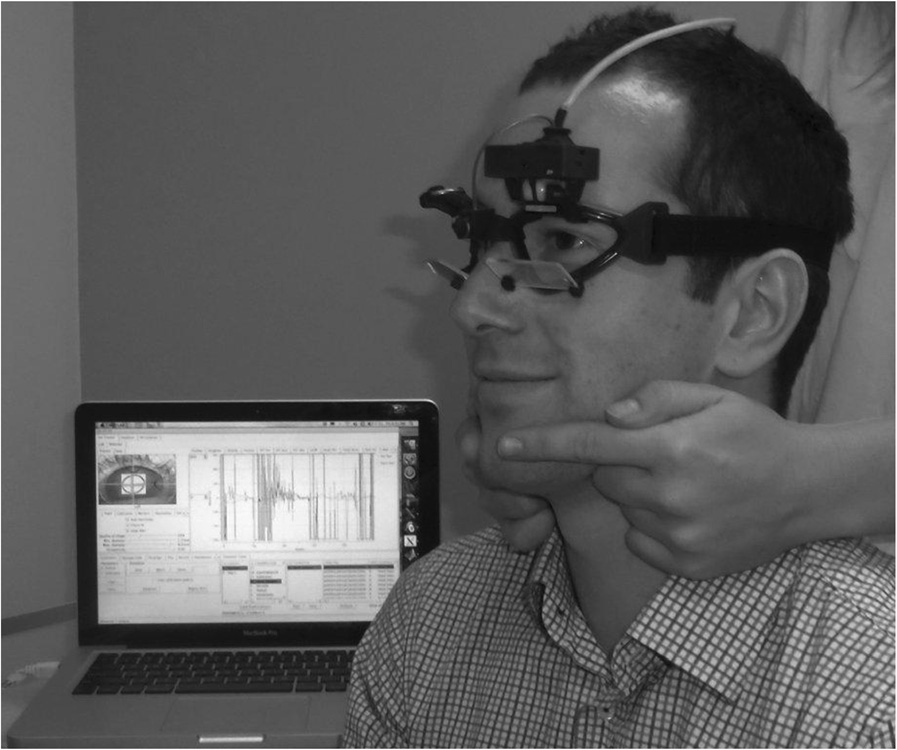
Method of testing, where the subject’s head is firmly held by the mandible, with three fingers clasped below and a thumb and forefinger above the jaw line

Eye and head rotations were measured during the HIT while the examiner manually applied rapid unpredictable (in direction and time) angular head rotations (peak head velocity 150 °/s to 300 °/s) [7]. Instantaneous HVOR velocity gains were calculated by the EyeSeeCam VOG software at 80 ms and 60 ms [8]. Head accelerations were manually controlled so that peak head velocity would occur at 80 ± 15 ms into each rotation [9]. This was achieved with angular head displacements of small amplitude (6° – 12°) and rapid rotation. Rotation of the subject’s head was performed with the examiner standing behind the seated subject. Six to ten unpredictable head rotations in both directions were performed from a central head position. This sequence was repeated twice to ensure adequate data collection and a check for data consistency, but more frequently if a subject needed additional training or if results were affected by artefact. Head rotations were achieved by the examiner firmly holding the mandible with three fingers clasped below and a thumb and forefinger above the jaw line (Fig. 1). This reduced skin movement and thus goggle slippage, decreasing the amount of artefact. Care was taken to avoid touching the goggles strap during head rotation. A single examiner^BM^ performed all tests throughout the study.

### Data analysis

**Fig. 2:**
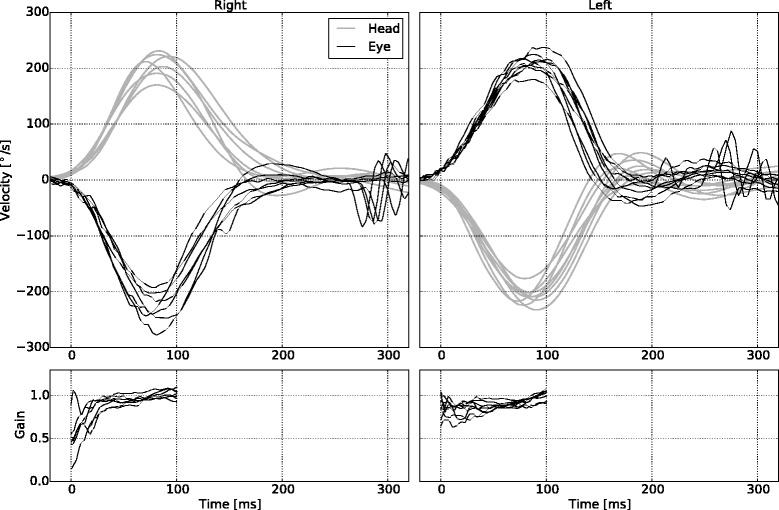
Example of selected data of HVOR velocity gain vs time, with no artefact

**Fig. 3:**
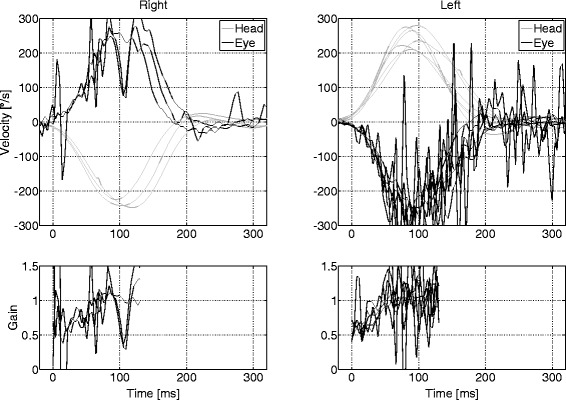
Example of omitted data of HVOR velocity gain vs time with artefact. In this subject, too few head rotations were plotted, and a narrow palpebral fissure lead to artefact. Nevertheless, the examiner can determine from the observation of the eye and head velocity traces that the vestibular function of this subject is normal. Despite the artefacts, one can observe that peak eye velocity corresponds with peak head velocity

Data with undue technical artefact (with blinks and obscured pupils) was discarded. This selection was unbiased and not determined by HVOR velocity gain. The graphed HIT sequences for each subject were assessed and the test with the least artefact was selected for analysis of HVOR velocity gain (Figs. 2 and 3 exemplify reliable and artefactual data). Only one of the HIT sequences was used in order to achieve similar properties to those expected from real-world clinical examinations. An average of 3.2 (range 2–6) HIT sequences for each subject were used to calculate repeatability of the test. This method took a pooled standard deviation for the left and right and used a definition for a repeatability coefficient adopted by the British Standards Institution [10].

HVOR velocity gain at 80 ms and 60 ms for both left and right rotations were used to assess the normative range. The 80 ms time analysis was used in case of artefact occurring in the early stages of head rotation (and attributed to goggle slip) and the 60 ms time analysis because of the frequent occurrence of covert catch up saccades seen in some patients with vestibular deficits. Two analysis intervals obviate these potential problems and provide an internal check for reliability. Analysis for each head impulse was performed over a 10 ms window centred at 80 ms and 60 ms. The median of these values was calculated in order to lessen the weighting from any outliers. Data was manipulated using the EyeSeeCam VOG software backed by Matlab scripts for data analysis. The same scripts were used in another study with search coils [8]. This analysis over a short time window has been recognised as the gold standard [2].

The main purpose of the study was to assess the relationship between HVOR velocity gain and age with analysis using a mixed regression model with a random effect for subjects. Differences between time of HVOR velocity gain analysis (80 ms or 60 ms) and sides were compared with analysis of variance with terms for time of velocity gain analysis, side, their interaction and their random terms with subjects.

Additional testing was also performed to assess the effect of fixation distance and predictable versus unpredictable head impulses on data reliability.

Five normal subjects were tested at a series of fixation distances (0.23, 0.40, 0.70, 1.00, 1.30, 1.60 and 1.90 m) to assess the dependence of the HVOR velocity gain on distance. This was analysed using a mixed linear regression with a random term for subject and autoregressive errors with distance.

Eighteen of the normal subjects were tested using both predictable and unpredictable head rotations (in direction and time). Predictable head impulses in direction alternated sequentially between the right and left at a regular time interval. The effect of predictable testing was analysed for variance with terms for side, whether or not the rotation was predictable, a random term for subjects, and interaction terms.

## Results

**Fig. 4:**
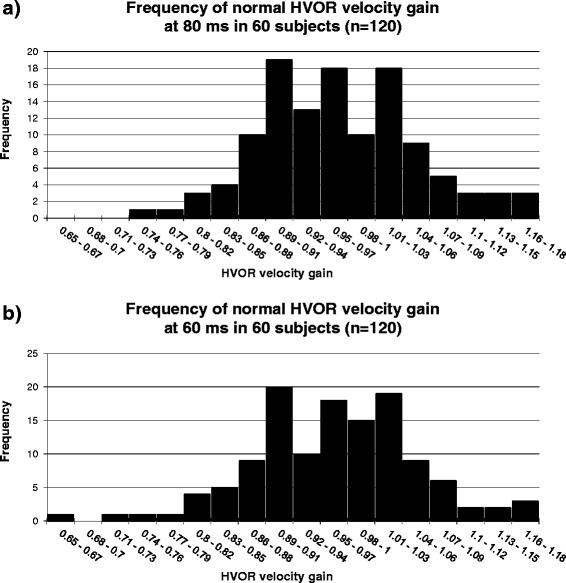
**a**: Normal HVOR velocity gain frequency at 80 ms across the second to eighth decades during left and right head rotations. Results show a normal distribution (Shapiro - Wilk test for normality, *p* = 39). HVOR velocity gain is the ratio of angular eye velocity to angular head velocity. **b**: Normal HVOR velocity gain frequency at 60 ms across the second to eighth decades during left and right head rotations. Results show a normal distribution (Shapiro - Wilk test for normality, *p* = 0.54). HVOR velocity gain is the ratio of angular eye velocity to angular head velocity

HVOR velocity gains were obtained for 63 subjects over the six decades from age 20 to 80. Horizontal head impulses were carried out with peak head velocities ranging from 150 °/s to 300 °/s (corresponding to peak head accelerations of 2300 °/sec^2^ to 5900 °/sec^2^). Results in three subjects (5 %) were discarded due to neck stiffness limiting angular head velocity, and artefact attributed to narrowed palpebral fissures (aged > 60 years). The remaining 60 subjects’ left and right HVOR velocity gains were plotted showing a frequency distribution. This included subjects with and without overt saccades as occur in normal subjects clinically. This gave a combined total of 120 HVOR velocity gains at both 80 ms and 60 ms, as displayed in Fig. 4a and b.

The distributions of HVOR velocity gain at 80 ms and 60 ms were not significantly different from normal distributions (Shapiro-Wilk test, W = 0.988, *p* = 0.39 and W = 0.990, *p* = 0.54 respectively) around mean values of 0.97 (SD = 0.09) and 0.94 (SD = 0.10), *n* = 120. The mean (95 % confidence intervals (CI)) HVOR velocity gains to the left and right are both 0.97 (0.94 – 0.99) at 80 ms, and 0.94 (0.92 – 0.96) to the left and 0.94 (0.92 – 0.97) to the right at 60 ms. The lower limit of the normal HVOR velocity gain (2SD below mean) was 0.79 at 80 ms and 0.75 at 60 ms. The lowest and highest values of the normal HVOR velocity gain were 0.76 and 1.18 at 80 ms and 0.65 and 1.17 at 60 ms.

The interaction of the time of analysis (80 ms or 60 ms) and side was not significant (*p* = 0.91). The HVOR velocity gains were significantly different between 80 ms and 60 ms (0.02; 95 %CI 0.04–0.01; SD 0.05; *p* = 0.0004). The HVOR velocity gains were not significantly different between the sides (0.00; 95 %CI -0.02–0.02; SD 0.08; *p* = 0.93). The HVOR velocity gain repeatability coefficients were 0.12 at 80 ms and 0.10 at 60 ms (95 % of differences are expected to be less than these).

**Fig. 5:**
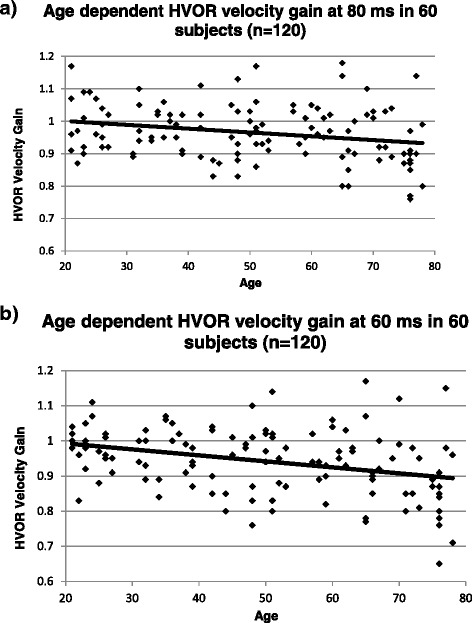
**a**: Normal HVOR velocity gain at 80 ms decline with age. HVOR velocity gain was found to decline by 0.012 per decade with increasing age (95 % CI 0.001 to 0.022; *p* = 0.028). HVOR velocity gain is the ratio of angular eye velocity to angular head velocity. **b**: Normal HVOR velocity gain at 60 ms decline with age. HVOR velocity gain was found to decline by 0.017 per decade with increasing age 95 %CI 0.006 – 0.029; *p* = 0.005). HVOR velocity gain is the ratio of angular eye velocity to angular head velocity

The HVOR velocity gain at 80 ms declined by 0.012 (95 % CI 0.001 – 0.022) per decade as age increased (*p* = 0.028), and at 60 ms declined by 0.017 (95 % CI 0.006 – 0.029) per decade as age increased (*p* = 0.005) (Fig. 5a, b). In patients younger than 70 years, the HVOR velocity gain was always above 0.80 at 80 ms and always above 0.76 at 60 ms.

The normalised HVOR velocity gain asymmetry was the absolute difference between left and right divided by the sum × 100 [11]; the 95^th^ percentile for 80 ms is 9.2 with a maximum of 12.5 and for 60 ms is 8.8 with a maximum of 16.7. There was no significant correlation with age at 80 ms or 60 ms with Spearman correlation coefficients of 0.12 (*p* = 0.36) and 0.11 (*p* = 0.42) respectively. The un-normalised gain asymmetry, or absolute value of the gain difference between the two sides [12, 13], was zero; the 95^th^ percentile for 80 ms is 0.17 with a maximum 0.26 and for 60 ms is 0.18 with a maximum 0.26.

During testing the direction and time of head rotation was unpredictable. In addition, eighteen of the subjects were also tested using a predictable HIT. The mean HVOR velocity gain at 80 ms was 0.06 (95 % CI 0.01 – 0.10; *p* = 0.014) higher when testing was carried out in a predictable manner (as high as 1.35). HVOR velocity gain between the left and the right increased similarly with predictable rotations (*p* = 0.55).

Five subjects were also tested for target fixation dependence of the HVOR velocity gain at 80 ms over increasing distance (0.23, 0.4, 0.7, 1.0, 1.3, 1.6 and 1.9 m). There was an inverse relationship between HVOR velocity gain and distance, with no significant difference between the rate of HVOR velocity gain change for the left and right (*p* = 0.12). With a common slope, the HVOR velocity gain decreased by 13 % (95 % CI 9 % – 17 %; *p* = 0.002) per metre.

## Discussion

In 60 normal subjects aged 20–80 years, the mean HVOR velocity gain at 80 ms of 0.97 was little different from that at 60 ms of 0.94. The 2 SD lower limit of the HVOR velocity gain at 80 ms was 0.79 and at 60 ms was 0.75. Although statistically significant, the difference in mean HVOR velocity gain, rounded to 0.02 (95 % CI 0.04–0.01) between 80 ms and 60 ms is not clinically important. Nevertheless, in patients with an impaired HVOR velocity gain, we think 60 ms is a more accurate point of measure in the presence of covert saccades than 80 ms. Nonetheless, using both points of measure provides a check on the consistency of results, and the flatness of the gain trajectory in normal subjects confirms the absence of slip (see lower half of Fig 2).

The lowest and highest values of HVOR velocity gain at 80 ms (0.76 and 1.18 respectively) were very close to those found in another study using a similar VOG camera system, with eight normal subjects (HVOR velocity gains of 0.75 and 1.2 respectively). Comparable results were found in the same eight subjects using search coils with a lowest and highest HVOR velocity gain of 0.70 and 1.00 [14]. That study analysed HVOR velocity gain over a 40 ms window centred at peak acceleration, while our analysis is over a 10 ms window. The mean HVOR velocity gain at 80 ms of 0.97 (SD = 0.09) and at 60 ms of 0.94 (SD = 0.10) for the 60 subjects also sits within a range of HVOR velocity gains observed using the search coil method with comparable accelerations [15].

Earlier studies using vHIT had not referenced the variation of normal HVOR velocity gain with respect to age [14], though recently VOR gain was found to decrease significantly in subjects older than 70 years [16] or 80 years [17]. In our study, HVOR velocity gain at 80 ms and 60 ms declined by 0.012 and 0.017 respectively per decade as age increased. This decline in HVOR velocity gain with age is consistent with the decline shown in a previous study of the HVOR velocity gain with head impulses using the search coil method [18]. At a practical level, the variation of the HVOR velocity gain with age both with vHIT and search coils was small, justifying the inclusion of our 10 subjects in the eighth decade in normative data, despite a statistically significant decrease in HVOR velocity gain with age. Unlike vestibular evoked myogenic potentials which may be lost with age, our results suggest that the vHIT HVOR velocity gain stands up relatively well with age.

Our highest HVOR velocity gain of 1.18 is not physiological and is likely to relate to goggle slip. This was minimised through firm placement of the examiner’s hands, clear from the goggle straps, holding the mandible to reduce slip. However, in the elderly who have looser skin and people with different facial structures or long hair, goggle slippage may still occur. We did not attempt to reduce slippage by using band aids across the nose, or placing dental paste on the nose [19], though these approaches might be considered if an individual subject’s slippage affects the interpretation of results.

An adequate head acceleration of 2300 °/sec^2^ to 5900 °/sec^2^ was achieved in each HIT to ensure the detection of a high frequency vestibular deficit [13, 18]. This was monitored in real time by ensuring a peak head velocity of 150 °/s to 300 °/s at 80 ms with low amplitude head movements.

Repeatability at 80 ms and 60 ms revealed consistent results across all four tests carried out on each subject. This verifies that in the absence of artefact, only one sequence of 6 – 10 sets of rotation in each direction is necessary for clinical interpretation of the HVOR.

It is important to ensure that the method of the HIT is unpredictable in direction and time. Testing in a predictable method confirmed a non-physiological abnormally high HVOR velocity gain. This may result from pre-programming which can augment the VOR and enhance the HVOR velocity gain [20, 21].

Analysis was carried out at a fixation target distance of 1.50 m. HVOR velocity gain depends on target distance. As target distance was decreased, HVOR velocity gain increased, consistent with findings during search coil testing [22, 23]. In our study, the major influence of target distance on HVOR velocity gain appeared to occur at distances of less than 0.70 m. Comparable HVOR velocity gains were found between our study (testing at a 1.50 m fixation target distance) and another study (testing at a 0.91 m fixation target distance) [14]. The dependency of the HVOR velocity gain on target distance is due to the different topography of the axes of rotation of the eyes and head [23].

VOG overcomes the clinical problem of a false negative HIT due to covert saccades and allows discrimination between physiological and pathological overt saccade(s). A clinical application is that in acute vertigo with clear impairment of HVOR velocity gain and the absence of CNS symptoms or signs, neuroimaging is not required; while the acutely vertiginous subject with a normal HVOR velocity gain needs consideration of a CNS cause or inferior vestibular neuritis [24]. The results of this study allow a comparison to be drawn between normal subjects and patients with vestibular lesions, across adult age groups to 80 years.

## Conclusions

The mean HVOR velocity gain of 60 normal subjects was 0.97 (SD = 0.09) at 80 ms and 0.94 (SD = 0.10) at 60 ms. Despite a significant variation in the HVOR velocity gain with age, these changes are minor, declining by 0.012 and 0.017 per decade as age increased at 80 ms and 60 ms respectively, justifying the same normative data for the second to the eighth decades. Normative data at 60 ms provides an opportunity for assessment when the 80 ms time interval result may be affected by the appearance of catch-up covert saccades.

## Abbreviations

HIT: Horizontal head impulse test
HVOR: Horizontal vestibulo-ocular reflex
vHIT: video HIT
VOG: Video-oculography
VOR: Vestibulo-ocular reflex
